# Early lesion detection with ^18^F-DCFPyL PET/CT in 248 patients with biochemically recurrent prostate cancer

**DOI:** 10.1007/s00259-019-04385-6

**Published:** 2019-06-22

**Authors:** M. Wondergem, B. H. E. Jansen, F. M. van der Zant, T. M. van der Sluis, R. J. J. Knol, L. W. M. van Kalmthout, O. S. Hoekstra, R. J. A. van Moorselaar, D. E. Oprea-Lager, A. N. Vis

**Affiliations:** 1 Noordwest Ziekenhuisgroep, Nuclear Medicine, Wilhelminalaan 12, 1815 JD Alkmaar, the Netherlands; 2 Amsterdam University Medical Centers, VU University, Urology, De Boelelaan 1117, 1081 HV Amsterdam, the Netherlands; 3 Amsterdam University Medical Centers, VU University, Radiology & Nuclear Medicine, Amsterdam, the Netherlands; 4 Radiology & Nuclear Medicine, University Medical Center Utrecht, Utrecht, the Netherlands

**Keywords:** ^18^F-DCFPyL PET/CT, Biochemically recurrent, Prostate cancer, PSMA

## Abstract

**Purpose:**

Prostate-specific membrane antigen (PSMA) PET/CT is increasingly used in patients with biochemically recurrent prostate cancer (BCR), mostly using gallium-68 (^168^Ga)-labelled radiotracers. Alternatively, fluorine-18 (^18^F)-labelled PSMA tracers are available, such as ^18^F-DCFPyL, which offer enhanced image quality and therefore potentially increased detection of small metastases. In this study we evaluate the lesion detection efficacy of ^18^F-DCFPyL PET/CT in patients with BCR and determine the detection efficacy as a function of their PSA value.

**Methods:**

A total of 248 consecutive patients were evaluated and underwent scanning with ^18^F-DCFPyL PET/CT for BCR between November 2016 and 2018 in two hospitals in the Netherlands. Patients were examined after radical prostatectomy (52%), external-beam radiation therapy (42%) or brachytherapy (6%). Imaging was performed 120 min after injection of a median dose of 311 MBq ^18^F-DCFPyL.

**Results:**

In 214 out of 248 PET/CT scans (86.3%), at least one lesion suggestive of cancer recurrence was detected (‘positive scan’). Scan positivity increased with higher PSA values: 17/29 scans (59%) with PSA values <0.5 ng/ml; 20/29 (69%) with PSA 0.5 to <1.0 ng/ml; 35/41 (85%) with PSA 1.0 to <2.0 ng/ml; 69/73 (95%) with PSA 2.0 to <5.0 ng/ml; and 73/76 (96%) with PSA ≥5.0 ng/ml. Interestingly, suspicious lesions outside the prostatic fossa were detected in 39–50% of patients with PSA <1.0 ng/ml after radical prostatectomy (i.e. candidates for salvage radiotherapy).

**Conclusion:**

^18^F-DCFPyL PET/CT offers early detection of lesions in patients with BCR, even at PSA levels <0.5 ng/ml. These results appear to be comparable to those reported for ^68^Ga-PSMA and ^18^F-PSMA-1007, with potentially increased detection efficacy compared to ^68^Ga-PSMA for patients with PSA <2.0.

**Electronic supplementary material:**

The online version of this article (10.1007/s00259-019-04385-6) contains supplementary material, which is available to authorized users.

## Introduction

Prostate cancer (PCa) is the most common cancer in men in the Western world [1, 2]. Initial therapy includes local intervention with curative intent, such as radical prostatectomy (RP), external-beam radiation therapy (EBRT), or brachytherapy (BT). However, between 28% and 53% of all such treated patients will develop biochemically recurrent prostate cancer (BCR) [3]. BCR is defined as two consecutive prostate-specific antigen (PSA) values ≥0.2 ng/ml after RP, or any PSA increase of 2.0 ng/ml above the nadir following EBRT and BT [4–6]. Accurate imaging studies are important for patients with BCR, as early lesion localisation directs further treatment, which might include stereotactic metastasis-directed radiotherapy, salvage radiotherapy, salvage lymph node dissection, or the initiation of systemic treatment [3].

Positron emission tomography/computed tomography (PET/CT) using radiotracers that bind to the prostate-specific membrane antigen (PSMA) is increasingly used for PCa diagnostics. PSMA is a class II transmembrane glycoprotein that provides a valuable target for radiolabelled imaging, as it is significantly overexpressed in malignant prostate cells [7]. So far, studies have primarily investigated gallium-68 (^68^Ga)-labelled PSMA tracers (^68^Ga-PSMA-HBED-CC), which have demonstrated promising results in patients with BCR [8]. Alternatively, fluorine-18 (^18^F)-labelled PSMA tracers are available, most notably ^18^F-DCFPyL [9, 10] and ^18^F-PSMA-1007 [11]. Because of a shorter positron range and higher positron yield, the ^18^F radionuclide provides higher PET image resolution than ^68^Ga, which may improve the detection of small metastases (e.g. at low PSA values) [12]. Although ^18^F-DCFPyL PET/CT is increasingly used in clinical practice, only minimal data are yet available on the diagnostic efficacy in patients with BCR [13]. Hence, the aim of this study was to determine the lesion detection efficacy of ^18^F-DCFPyL PET/CT in patients with BCR and establish the efficacy as a function of patients’ current PSA values.

## Material and methods

This retrospective analysis consists of 248 patients with BCR consecutively scanned with ^18^F-DCFPyL PET/CT from November 2016 until December 2018 in two Dutch hospitals (Amsterdam University Medical Centres, VU University; Noordwest Ziekenhuisgroep, Alkmaar). Patients with BCR were included regardless of prior curative treatment (RP, EBRT, BT). Patients with persistent PSA after curative treatment or patients with castration-resistant prostate cancer were excluded. If multiple ^18^F-DCFPyL PET/CT scans for BCR were performed for a patient, only the first examination was included. No exclusion criteria were deployed other than the coexistence of other malignancies. Patient characteristics are presented in Table 1.

**Table 1:** Patient characteristics

Characteristics		*n = 248*
Age, years (median, IQR)		71 (67–75)
		*n* (%)
PSA at PET/CT (ng/ml)	<0.5	29 (12%)
	0.5–1	29 (12%)
	1–2	41 (17%)
	2–5	73 (29%)
	>5	76 (31%)
PSA doubling time (months) (median, IQR)		6 (3–12)
Gleason score	6	33 (13%)
	7	97 (39%)
	8	36 (15%)
	9–10	48 (19%)
	Unknown	34 (14%)
Tumour stage	T1c	16 (6%)
	T2	82 (33%)
	T3	109 (44%)
	T4	7 (3%)
	Unknown	34 (14%)
Initial therapy	Radical prostatectomy	128 (52%)
	External-beam radiation	105 (42%)
	Brachytherapy	15 (6%)
ADT at PET/CT		20 (8%)
Prior salvage radiation therapy		41 (17%)

IQR = interquartile range

## Imaging

^18^F-DCFPyL was synthesised under good manufacturing practice (GMP) conditions at the on-site cyclotron facilities of both hospitals [14]. PET acquisitions were made 120 min after injection of a median dose of 311 MBq ^18^F-DCFPyL (interquartile range 284–325 MBq). Imaging was performed with a Philips Ingenuity TF (Philips Healthcare, the Netherlands/USA) and a Siemens Biograph TruePoint-16 (Siemens Healthineers, Germany) PET/CT scanner. The scan trajectory included mid-thigh to skull vertex, with 4 min (Philips scanner) and 5 min (Siemens scanner) per bed position. PET acquisitions were combined with a low-dose CT or contrast-enhanced CT scan (30–110 mAs, 110–130 kV). All images were corrected for decay, scatter, and random coincidences; photon attenuation correction was performed using CT images.

Images were reconstructed with the vendor-provided BLOB-based ordered-subset expectation maximisation algorithm on the Philips system (3 iterations; 33 subsets) [15] and the ordered-subset expectation maximisation algorithm on the Siemens system (4 iterations; 16 subsets, including a 5-mm Gaussian filter). The reconstructed images had a maximum image matrix size of 256 × 256, voxel size 2.67 × 2.67 × 4 mm (Siemens data) and matrix size 288 × 288, voxel size 2 × 2 × 2 mm (Philips data).

### Image interpretation

Scan interpretation was performed in the participating centres by four nuclear medicine physicians in total, with ample experience in PCa PET reading (>200 scans). Dual-reading was performed for all scans, the final conclusion was drawn up in consensus, recording the localisation of detected lesions (i.e. prostate/prostatic fossa, loco-regional lymph nodes, distant lymph nodes, bones, visceral organs). A scan was considered ‘positive’ if at least one lesion suggestive of PCa recurrence was detected. Prostate lesions and lymph nodes were considered positive when the activity in those lesions exceeded blood pool activity. Bone lesions were considered positive if the activity was higher than general bone marrow activity, without CT findings clearly demarcating benign lesions such as hemangioma.

### Statistical analysis

Numerical variables were summarised as medians and interquartile ranges; categorical variables with proportions (%). Scan positivity was calculated for the following PSA strata (<0.5; 0.5 to <1.0; 1.0 to <2.0; 2.0 to <5.0; ≥5.0 ng/ml) and includes a 95% confidence interval (CI). Binary logistic regression analyses were performed to identify predictors of scan positivity (e.g. PSA value at the time of PET/CT, PSA doubling time, Gleason score, tumour stage, use of androgen deprivation therapy (ADT) at the time of PET/CT). Differences in the distribution of detected lesions (e.g. local recurrence, regional lymph nodes) between PSA strata were tested using Fisher’s exact test with Holm–Bonferroni correction.

## Results

**Fig. 1:**
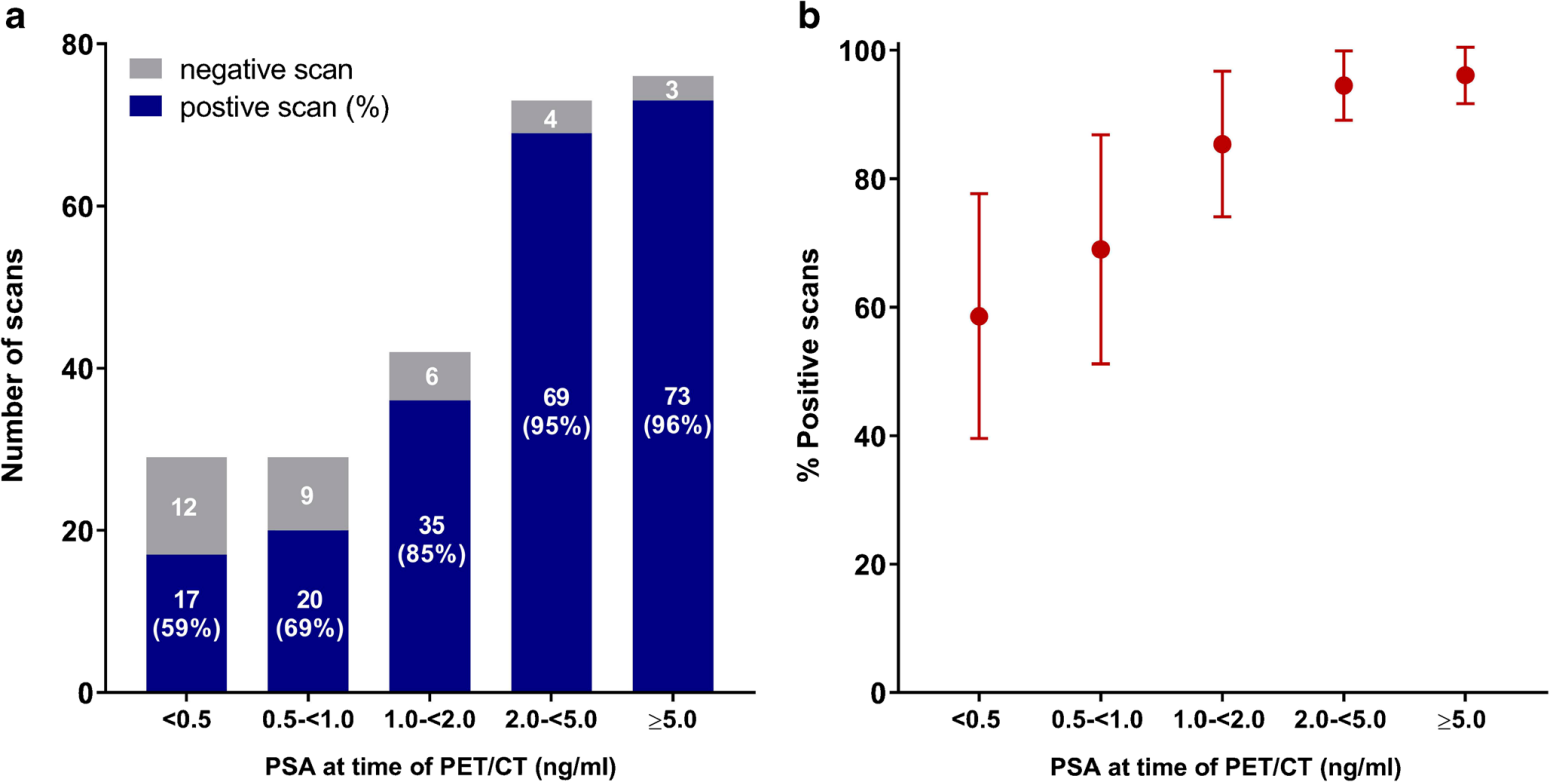
(**a**) Detection efficacy of ^18^F-DCFPyL PET/CT in patients with BCR and (**b**) the proportion of positive scans (including 95% confidence intervals)

**Fig. 2:**
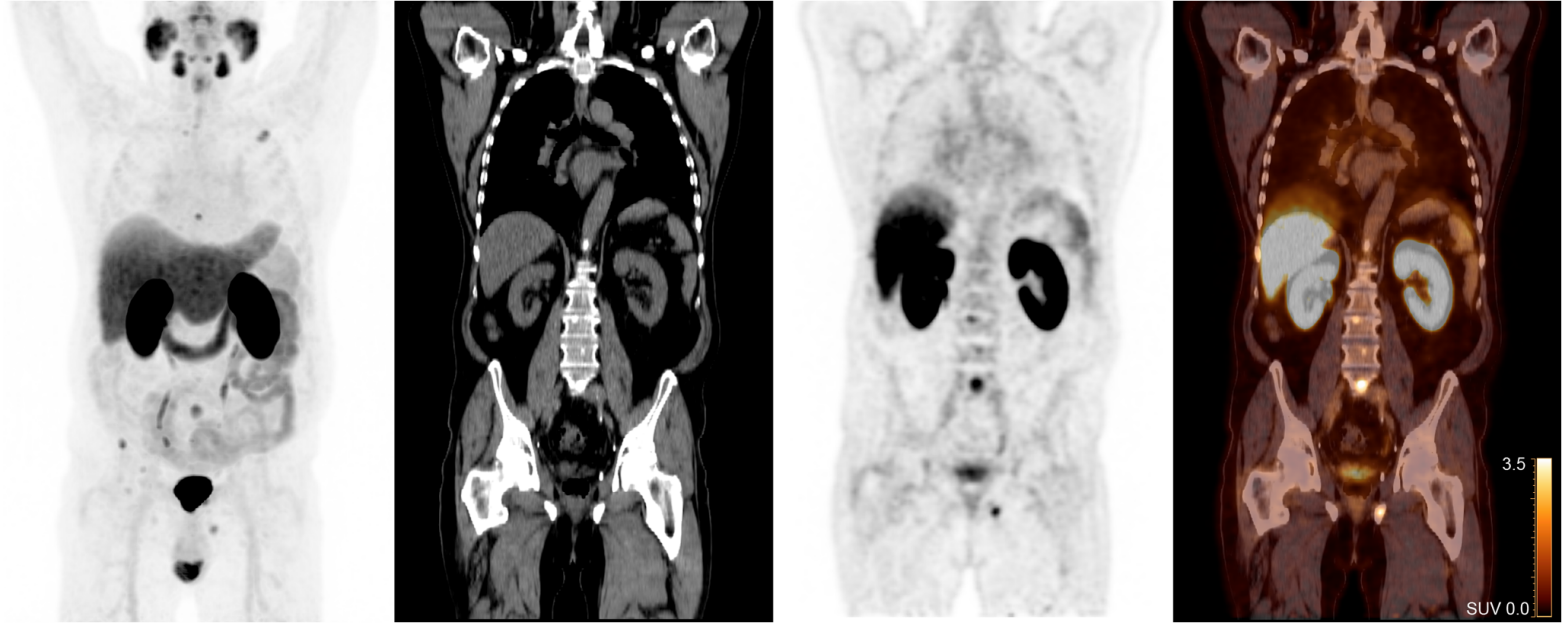
Illustration of detection of bone metastases in a patient with BCR after RP and low PSA value (0.7 ng/ml). Left: maximum-intensity projection. Right: coronal CT, PET and fusion images

In 214 out of 248 ^18^F-DCFPyL PET/CT scans (86.3%, 95% CI 82.0–90.6%), at least one lesion suggestive of PCa recurrence was detected (positive scan). Lesion detection increased with higher PSA values, with a detection efficacy of 59% (17/19 scans), 69% (20/29), 85% (35/41), 95% (69/73) and 96% (73/76), respectively, for scans at PSA values <0.5, 0.5 to <1.0, 1.0 to <2.0, 2.0 to <5.0 and ≥ 5.0 ng/ml (Figs. 1, 2). Excluding patients with incomplete clinical history (e.g. Gleason score, see Table 1) for the evaluation revealed similar results (Supplementary data 1). Aside from current PSA value, no other parameter (Gleason score, tumour stage, current ADT use, PSA-doubling time, previous salvage radiotherapy) was a significant predictor of ^18^F-DCFPyL PET/CT scan positivity in regression analysis (Supplementary data 2).

**Fig. 3:**
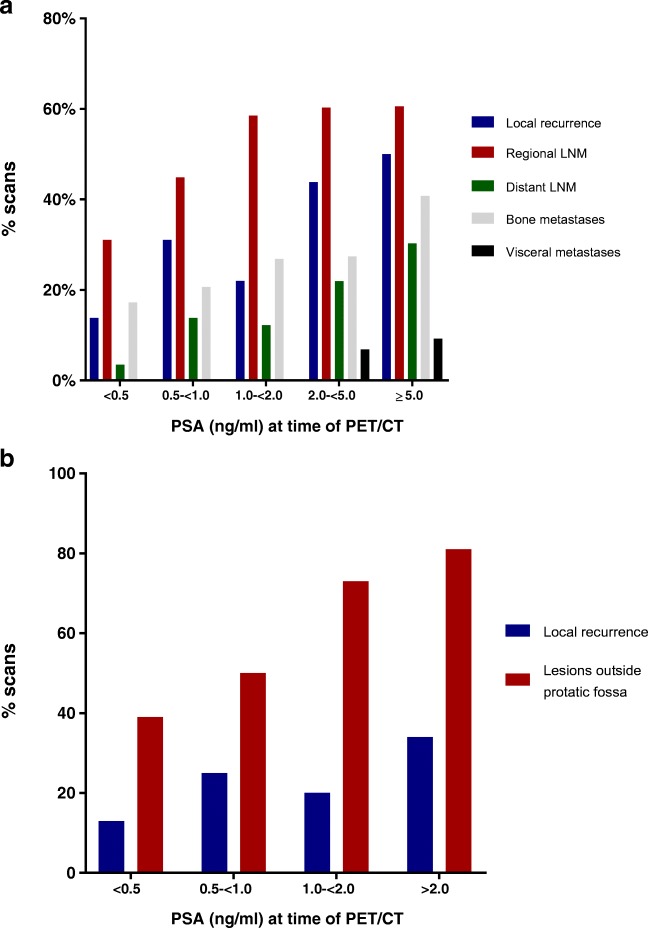
(**a**) Characteristics of the detected lesions in all patients with BCR (**b**) Characteristics of detected lesions in patients with BCR after radical prostatectomy, not yet treated with salvage radiotherapy

Scans were most frequently positive due to detection of regional lymph node metastases (136/248 scans, 54.8%), followed by local recurrence in the prostate or prostatic fossa (92/248 scans, 37.1%); bone metastases (73/248, 329.4%); distant lymph node metastases (49/248, 19.8%); and visceral metastases (12/248, 4.8%) (Fig. 3, Supplementary data 3). The relative distribution of detected lesions was not significantly different at the various PSA strata (*p* = 0.22 to > 0.9, Supplementary data 4).

A subgroup analysis was performed for patients with BCR after RP and low PSA values (<1.0 ng/ml) (*n* = 43), as they may be candidates for curative salvage radiotherapy to the prostate bed [3]. In our cohort, ^18^F-DCFPyL PET/CT revealed suspicious lesions outside of the prostatic fossa in 39–50% of these patients (Figs. 2, 3).

**Fig. 4:**
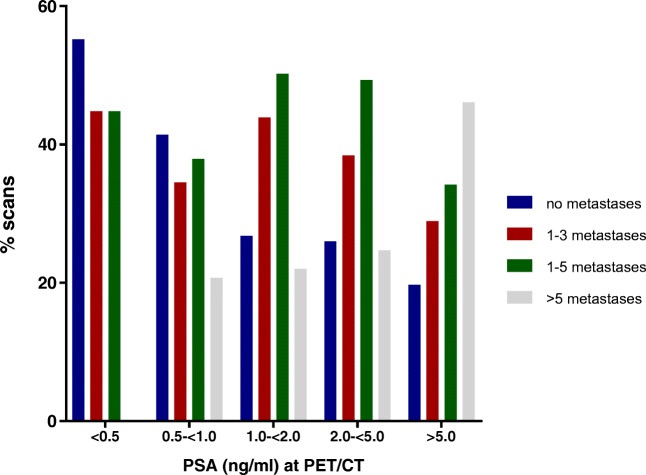
Number of detected lesions per PSA stratum. Red and green bar indicating patients with oligometastases (defined as either ≤3 or ≤ 5 metastases, with or without a local recurrence). Grey bar representing patients with polymetastases (>5 metastases)

All patients who underwent ^18^F-DCFPyL PET/CT with a PSA <0.5 ng/ml revealed ≤3 metastases (‘oligometastatic disease’, Fig. 4). Conversely, in patients with a PSA ≥5.0 ng/ml, only 29% (22 out of 76) were diagnosed with 1–3 metastases (34% with 1–5 metastases) (Fig. 4).

## Discussion

In this study we evaluated the lesion detection efficacy of ^18^F-DCFPyL PET/CT in 248 patients with BCR. Suspected metastases were identified in the majority of PET examinations and even at PSA values <0.5 ng/ml, lesions were observed in 59% of patients. These results show clear improvement over the results with conventional imaging modalities, as well as choline PET/CT [16–18]. Compared to a recent meta-analysis on ^68^Ga-PSMA PET/CT [8], our study demonstrates equivalent results in patients with PSA values >2.0 ng/ml (i.e. 95% lesion detection with both tracers). Potentially improved detection is observed compared to ^68^Ga-PSMA in all PSA strata <2.0 ng/ml (most notably, 59% detection with ^18^F-DCFPyL when PSA <0.5 ng/ml vs. 45% with ^68^Ga-PSMA; Table 2) [8].

**Table 2:** Comparison of results of various PSMA radiotracers

Publication	Tracer	*N*	Detection rates per PSA strata (ng/ml)			
			<0.5	0.5 to <1.0	1.0 to <2.0	≥2.0
Perera 2019 [8]	^68^Ga-PSMA-11	4790	45%	59%	75%	95%
			<0.5	0.5 to <1.0	1.0 to <2.0	≥2.0
Giesel 2018 [24]	^18^F-PSMA-1007	251	62%	74%	91%	94%
			≤0.5	0.51 to ≤1.0	1.1 to ≤2.0	>2.0
Rahbar 2018 [25]	^18^F-PSMA-1007	100	86%	89%	100%	100%
			<0.5	0.5 to <1.0	1.0 to <2.0	≥2.0
*Present study* 2019	^18^F-DCFPyL	248	59%	69%	85%	96%

Recently revised European Association of Urology guidelines recommend performing PSMA PET/CT at BCR after RP if the PSA level is >0.2 ng/ml and if the results will influence subsequent treatment decisions [19]. PSMA PET/CT is recommended in case of BCR after EBRT in patients fit for curative salvage treatment. The guidelines also recommend offering a PSMA PET to men with a persistent PSA >0.2 ng/ml after prostatectomy to exclude metastatic disease. Those recommendations are in line with our findings that even at the lowest PSA values, sites of PCa recurrence could be detected with ^18^F-DCFPyL PET/CT in the majority of patients.

Further, ^18^F-DCFPyL PET/CT revealed lesions outside the prostatic fossa even at very low PSA levels (e.g. 39% with PSA <0.5 ng/ml) (Figs. 2, 3). These findings are clinically relevant, because patients with BCR after RP are candidates for local salvage radiation treatment to the prostatic fossa, and such treatment is likely to be ineffective in the presence of (lymph node) metastases. Indeed, Emmett et al. recently demonstrated that ^68^Ga-PSMA PET/CT may be effective for stratification of patients into groups with a high probability of response to salvage radiotherapy (negative PET or detection of local recurrence only) versus lower probability of response (detected lesions outside the prostatic fossa). In that study (*n* = 164), PSMA PET findings predicted salvage therapy outcomes better than established predictors, most notably current PSA values [20].

To our knowledge, there is only one prior study on ^18^F-DCFPyL PET/CT for patients with BCR, analysing *n* = 62 ^18^F-DCFPyL PET/CT scans in direct comparison with ^68^Ga-PSMA PET/CT [13]. Dietlein et al. [13] found higher sensitivity with ^18^F-DCFPyL in patients with PSA values between 0.5–3.5 ng/ml (88% with ^18^F-DCFPyL versus 66% with ^68^Ga-PSMA, *p* = 0.042). Compared to Dietlein et al., we observed a higher detection efficacy, especially at PSA values <0.5 ng/ml (59% versus 13%, respectively). This difference might be due to the image interpretation protocol applied by Dietlein et al., which required corresponding findings on CT for all PET-positive lesions. Based on current standardised reporting systems, such a requirement is no longer applied for PSMA PET interpretation at our institutions [21, 22].

Recently, another ^18^F-labelled PSMA tracer was introduced: ^18^F-PSMA-1007 [23]. ^18^F-PSMA-1007 is only minimally excreted via the urinary tract, which potentially improves visualisation of tumour deposits adjacent to the urinary bladder (mainly local recurrence). Our results with ^18^F-DCFPyL PET/CT appear comparable to the outcomes of 251 ^18^F-PSMA-1007 PET/CT scans analysed by Giesel et al. (e.g. 62% positive scans with ^18^F-PSMA-1007 when PSA <0.5 ng/ml [24]) (Table 2). Conversely, Rahbar et al. report notably more positive ^18^F-PSMA-1007 PET/CT scans compared to both Giesel et al. and the present study (86% positive scans when PSA <0.5 ng/ml; 100% positive scans when PSA >1.0 ng/ml [25]). Rahbar et al. included only 100 patients, however, and may have included more advanced PCa cases. For example, 38% of RP patients analysed by Rahbar et al. had already received salvage radiotherapy (compared to 17% in our cohort), and 27% of patients received ADT around the time of examination (compared to 8% in our cohort). In general, precise comparison of the results with different PSMA tracers (including ^68^Ga-PSMA) is difficult. Most studies are retrospective, and many factors other than the applied radiotracers may have influenced the diagnostic results (e.g. included patient population, PET scanner technique, image reconstruction methods, experience of the PET readers). In particular for cohorts including patients treated with EBRT, such as our cohort, somewhat higher detection rates are generally found in comparison with populations treated with RP only.

In terms of detection of local recurrence, no evident benefit of ^18^F-PSMA-1007 over ^18^F-DCFPyL is seen. Giesel et al. included only patients with BCR after RP and detected local recurrence in 25% of patients. Rahbar et al. noted local recurrence in 37% of all patients (treated with either EBRT or RP) [24, 25]. In our study, local recurrence was detected in 24% of all patients treated with RP and in 37% of the entire population.

Contrary to our finding that PSA at PET/CT is the only predictor of scan positivity, a recent study by Rauscher et al. found that concurrent ADT was also a predictor of a positive scan in multivariable analysis [26]. It should be noted that in our cohort, only 20 patients were receiving ADT at the time of PET/CT, the majority of whom received ADT in combination with EBRT; Rauscher et al. included only patients after RP, all with a PSA <1.0 ng/ml. Overall, the role of ADT in scan outcomes remains incompletely understood, for ADT may upregulate PSMA expression initially [27], but reduce lesion detection efficacy upon longer exposure [28]. Patients who received ADT after prostatectomy due to known metastatic disease without salvage options—in other words, in a palliative setting—were not included in this cohort. Although there is no definition as to where BCR ends in the course of prostate cancer progression, in our opinion, a cohort of patients with BCR should only include patients who are candidates for salvage options before PSMA PET/CT is performed.

Accurate identification of patients with oligometastatic disease is of current interest [29], although the definition of oligometastatic disease remains unclear (either ≤3 or ≤5 metastases). The clinical benefit of (oligo)metastases-directed treatment has not yet been established [3]. Nevertheless, it seems evident that any success of metastases-directed strategies will depend on accurate imaging studies, as well as adequate timing of diagnostic procedures. From our data it can be observed that performing ^18^F-DCFPyL PET/CT soon after diagnosis of BCR (i.e. at lower PSA values) favours detection of oligometastases (Fig. 4). At higher PSA levels (>5.0 ng/ml), more patients are diagnosed with polymetastases (>5). Yet even in these patients, a substantial proportion had oligometastastic disease on ^18^F-DCFPyL PET/CT (Fig. 4).

An important limitation of the present study, and studies on PSMA PET for BCR in general, is the lack of histopathologic confirmation of PET results [8, 12, 30]. PSMA PET-detected lesions are often smaller than 1 cm, making biopsy procedures difficult and burdensome for patients. In our study, histologic confirmation of PET findings (CT-guided biopsy, lymph node dissection, prostate biopsy) had been performed in only 15 of our patients, although follow-up of patients from outside our PET centres was often lacking. In 12 of these patients (80%), the malignant nature of detected lesions was confirmed. Overall, these numbers are insufficient to assess the extent of false-positive outcomes (the specificity) in our study. High specificity of PSMA PET has been demonstrated in primary PCa patients, however, where imaging findings can be correlated to histopathology from lymph node dissection (96% specificity with ^68^Ga-PSMA [30]; 96–99% specificity with ^18^F-DCFPyL [31]). We were also unable to evaluate the sensitivity of ^18^F-DCFPyL on a lesion basis, since the true number of PCa metastases remains unknown. PET is inherently limited by its image resolution to the detection of small metastases (<2–4 mm). We should therefore assume that ^18^F-DCFPyL PET/CT still underestimates the extent of disease.

Clinically, it is important to realise that improved detection of metastases is only of benefit to patients if followed by appropriate, proven-effective therapeutic strategies. The identification of additional metastases may delay treatment of local recurrences, trigger metastasis-directed treatment, or result in initiation of systemic therapy. None of the clinical outcomes of these (PSMA PET-based) decisions have yet been sufficiently studied in clinical trials [3, 29, 32].

## Conclusion

^18^F-DCFPyL PET/CT appears effective for detecting metastases in patients with BCR, even at PSA values <0.5 ng/ml. The detection efficacy appears at least comparable to published results with ^68^Ga-PSMA and ^18^F-PSMA-1007.

## Electronic supplementary material

ESM 1 (DOCX 36 kb)
