# Supplementary material for: Early lesion detection with 18F-DCFPyL PET/CT in 248 patients with biochemically recurrent prostate cancer
Source: Eur J Nucl Med Mol Imaging. 2019 Jun 22;46(9):1911–8. doi: 10.1007/s00259-019-04385-6 (PMC6647179; doi:10.1007/s00259-019-04385-6)
Supplement: Supplementary file 1 — (DOCX 36 kb) [file 259_2019_4385_MOESM1_ESM.docx]

**Supplementary material**

**Supplementary data 1:** BCR localization rates excluding patients with incomplete basic patient characteristics

|  |  | *n* positive scans / total *n* scans | % |
| --- | --- | --- | --- |
| Total | | 175/203 | 86.2% |
|  |  |  |  |
| PSA (ng/ml) | <0.5 | 16/26 | 61.5% |
|  | 0.5-<1.0 | 16/25 | 64.0% |
|  | 1.0-<2.0 | 29/35 | 82.9% |
|  | 2.0-<5.0 | 61/62 | 98.4% |
|  | ≥5 | 53/55 | 96.4% |

**Supplementary data 2:** Results of univariable analysis predicting ^18^F-DCFPyL PET/CT positivity.

| Variable | Categories | OR | 95% CI | *p*-value |
| --- | --- | --- | --- | --- |
| PSA (ng/ml) | *(continuous)* | 1.44 | (1.16-1.8) | 0.001 |
|  |  |  |  |  |
| PSA (ng/ml) | <0.5 | *(ref)* |  |  |
|  | 0.5-1 | 1.57 | (0.53-4.62) | 0.414 |
|  | 1-2 | 4.12 | (1.32-12.86) | 0.015 |
|  | 2-5 | 12.18 | (3.49-42.49) | 0.000 |
|  | >5 | 17.18 | (4.36-67.65) | 0.000 |
|  |  |  |  |  |
| PSA doubling time (months) | | 0.99 | (0.88-1.10) | 0.599 |
|  |  |  |  |  |
| Gleason score | 6 | *(ref)* |  |  |
|  | 7 | 0.70 | (0.22-2.26) | 0.549 |
|  | 8 | 1.52 | (0.31-7.35) | 0.605 |
|  | 9-10 | 0.97 | (0.25-3.73) | 0.959 |
|  |  |  |  |  |
| Tumour-stage | T1c | *(ref)* |  |  |
|  | T2 | 0.32 | (0.04-2.66) | 0.294 |
|  | T3a | 0.67 | (0.07-5.96) | 0.717 |
|  | T3b-T4 | 0.41 | (0.05-3.61) | 0.421 |
|  |  |  |  |  |
| ADT at PET/CT | | 1.48 | (0.33-6.71) | 0.612 |
| prior Salvage Radiotherapy *^a^* | | 3.09 | (0.99-9.63) | 0.052* |
| EBRT versus RP *^b^* | | 1.29 | (0.28-6.03) | 0.744 |
| Hospital / PET system | | 0.49 | (0.23-1.04) | 0.063* |

*^a^* Only patients with BCR after RP were analysed.
*^b^* Only patients with PSA ≥2.0ng/ml were included (definition of BCR in patient treated with EBRT).
* Neither a significant predictor in multivariable analysis including PSA (results not shown).

**Supplementary data 3:** Characteristics of detected lesions, per initial treatment modality.

**Supplementary data 4:** Relative distribution of types of lesions at the different PSA strata (frequency of lesions detected in positive scans).
